# Stakeholder's perception of the health information system performance in Burkina Faso

**DOI:** 10.11604/pamj.2023.44.155.35294

**Published:** 2023-03-31

**Authors:** Cheick Omar Diallo, Karin Linda Schiøler, Koine Maxime Drabo, Helle Samuelsen

**Affiliations:** 1 Health sciences doctoral school, University Joseph Ki-Zerbo, Ouagadougou, Burkina Faso,; 2 Department of Public Health, University of Copenhagen (UCPH), Copenhagen, Denmark,; 3 Department of Public Health, University Joseph Ki-Zerbo/IRSS, Ouagadougou, Burkina Faso,; 4 Department of Anthropology, University of Copenhagen (UCPH), Copenhagen, Denmark

**Keywords:** Perception, performance, health information system, Burkina Faso

## Abstract

**Introduction:**

the health information system (HIS) in Burkina Faso has improved significantly in recent years. In order to suggest further improvements, we specifically assessed the HIS performance indicators of the epidemic surveillance system from the perspectives of the stakeholders.

**Methods:**

we conducted a mixed methods study to assess the performance through timeliness and completeness indicators, strengths, and weaknesses of the HIS in Burkina Faso with specific focus on epidemic surveillance in the health districts of Dandé and Tenkodogo for the period of 2016 to 2019.

**Results:**

fewer than 35% of health districts were able to report at least 90% completeness of community reports since 2017. In 2018, four districts did not exceed 1% completeness of community reports. Some concerns remain related to a need of local support and inter-sectoral collaboration. The technical and organizational factors affect process and performance of the system directly or indirectly through behavioral determinants.

**Conclusion:**

the ability to measure the performance of all health facilities and to share all community reports online are challenges for the health system in Burkina Faso. New technologies, training-sensitization, and the involvement of actors with influence on social or behavioral change could help to ensure dynamic performance, if perceptions of actors are taken into account.

## Introduction

According to the World Health Organization (WHO), a well-functioning Health Information System (HIS) is one that ensures the production, analysis, dissemination and use of reliable and timely health information [1]. It also ensures the availability of information to guide action and assess the functioning of the health system. Indeed, a good HIS encourages the provision of dynamic and efficient health services [2].

Most countries in sub-Saharan Africa have improved their HIS considerably after the Ebola outbreak in West Africa in 2014-2016 [3,4], mainly through an increased focus on digitalization and the use of information and communications technology. However, in many cases, the HIS is designed to meet the information needs of the central level of the health system using an administrative and hierarchical logic [5,6] that may result in missed opportunities to address operational needs at the local level.

In 2010, the national HIS strategic plan of Burkina Faso identified a number of weaknesses, including poor coordination of the entire national HIS, and the inadequacy of the system to motivate the staff in charge of health information [7]. It also identified redundant data collection tools, inadequate data collection at the community level, poor data harvesting as well as low representativeness of available data, as only 40% of the population attended health facilities [7].

Since then, the HIS of Burkina Faso has improved significantly, most notably through the introduction of an electronic health management information system (HMIS) in 2013 [8]. This system includes the collection and integration of routine health data across most health programs based on the District Health Information Software 2 (DHIS2) [8]. In spite of the recent HMIS efforts, the performance of the routine health information system (HIS) remains difficult to monitor given the lack of baseline data [8]. This includes the performance of the RHIS in relation to epidemic surveillance. Moreover, few efforts have been directed at understanding possible enablers and barriers to the routine processes, as perceived by those directly involved in the surveillance of epidemic diseases in Burkina Faso.

The objective of this study was to assess the performance of the RHIS of Burkina Faso with respect to epidemic surveillance at the health district (HD) level and to explore the strengths and weaknesses of the surveillance system as perceived by relevant stakeholders within the HIS.

## Methods

**Study design:** we conducted a convergent mixed methods study in the health districts of Dandé and Tenkodogo comparing the observed performance of the routine HIS with the strengths and weaknesses of the reporting system as perceived by those directly involved. Specifically, we assessed the performance of the HIS in a cross-sectional study evaluating the timeliness and completeness of surveillance reports submitted to and from the district level. At the same time, we used a phenomenological approach to explore the strengths and weaknesses of the HIS, as perceived by selected stakeholders engaged in epidemic surveillance at the health district level.

**Study sites:** situated in the West region of Burkina Faso, the health district of Dandé is on the border of Mali, whereas Tenkodogo, located in the East region, is close to Togo and Ghana. Given the continuous cross-border movement of humans, animals, and goods, the two districts were considered at increased risk of epidemic disease transmission and thus purposely selected for performance analysis of the HIS with respect to epidemic surveillance. Primary health care centers (PHCs), Soumagou (Tenkodogo) and “Vallée du Kou” (Dandé) were randomly selected among all PHCs in the two Districts.

**Data collection:** secondary data were retrieved from the central level database at the Health data warehouse (EnDoS/DHIS2) including the District/ Regional reports covering the period of 2016 to 2019 across all 15 HDs, including Dandé in Hauts Bassins (N=8) and Tenkodogo in Centre-East (N=7) regions. Supplementary data were collected from the national HIS data manager and corresponding statistical yearbooks [9-12]. In addition, we retrieved data from the university hospital of Bobo (CHU) and the regional hospital center (CHR) of Tenkodogo. Data included all weekly official letter telegrams (*Télégramme-lettre officiel hebdomadaire* (*TLOH*)) from 2016 to 2019, and all Monthly Activity Reports (MARs) from the health facilities as well as Community-Based Health Workers' (CBHW) reports. We referred to technical surveillance guides for the identification, notification and management of disease cases with epidemic potential [13-15]. Information relating to perceived strengths and weaknesses of the HIS was collected through semi-structured interviews of stakeholders associated with PHCs in Soumagou (Tenkodogo) and “Vallée du Kou” (Dandé). Informants included CBHWs as well as health workers involved in epidemic surveillance, such as the head nurse (*Infirmier chef de poste* (*ICP*)) at each PHC. Community members attending the PHC as patients were also included as were all members of the district, regional and central management teams.

**Data analysis:** we measured the performance of the routine HIS according to the timeliness and completeness of reported health data. Timeliness was defined as the percentage of expected reports delivered on time and completeness as the percentage of expected reports delivered during a given period [16]. The accepted national standard for both timeliness and completeness was 100% [16]. We used a document analysis guide to assess the timeliness and completeness of i) the transmission of data from Districts to the central level (TLOHs ), ii) the transmission of community reports from Districts to the central level (EnDoS/DHIS2), and iii) the transmission of MARs from Districts to the central level. We conducted a thematic analysis of all semi-structured interviews using the Performance of the Routine Information System Management (PRISM) framework, proposed by Aqil *et al*. Specifically, we assessed the strengths and weaknesses of the HIS as perceived by stakeholders according to the i) use of tools (technical factors), ii) coordination and organization of surveillance activities and impact of the workload on other activities (organizational factors), and iii) information and decision-making dynamics (behavioral factors) [17]. The findings from the two study components were compared for corroborations and/or disagreements.

**Ethical considerations:** informed consent was obtained for all study participants and anonymity was ensured during data management and analyses by assigning codes to each respondent before the transcription of interviews. The study was approved by the Ministry of Health and ethical clearance was provided by the institutional ethics committee of “Institut de la recherche en sciences de la santé” (IRSS), Burkina Faso. The study was registered under the number 32-2019/CEIRES on October 02, 2019.

## Results

**Descriptive statistics:** this study identified a total of 34 PHCs and one medical center (MC) in Dandé, as well as 26 PHCs and one MC in Tenkodogo for which reported health data were retrieved across 15 health districts. We interviewed 60 health professionals and community members (Table 1) distributed across the community (52%), the operational or decentralized district level (31%) as well as the intermediate and central levels (17%). The mean years of professional experience at the facility or in the job were 7.6 years (variations between 0.17 and 25 years) for health workers from PHCs to the central level and 5.5 years for community members (variations between 0.08 and 18 years). Moreover, 18% (11/60) of all interviewees were female. All health professionals and CBHWs declared that they were trained at least once in integrated disease surveillance and response, integrated disease surveillance and response (IDSR), by the Ministry of Health.

**Table 1:** distribution of interviewees (stakeholders) by profile and institutional level in the Centre-East and Hauts Bassins Regions, Burkina Faso

Institutional levels	Profiles	N = 60	% (of level)
**Central and regional management team**	DRS’s director	10	91
	Disease control service manager (SLM)		
	*Centre d’information sanitaire et de surveillance épidémiologique’s* officers (CISSE)		
	Data manager		
**District management team**	District medical officers (MCD)	9	100
	District CISSE		
	Other members		
**Primary health care centers**	*Infirmier chef de poste* (ICP)	10	100
	Health workers		
**Community**	Community-based health workers (CBHWs)	21	95
	Community-based organization (CBO) animators	2	-
	Community-based organization (CBO) monitoring-evaluation officers	2	-
	School teacher	1	-
	Village development advisors	5	-

Community-based health workers (CBHW), Community-based organization (CBO), Centre d’information sanitaire et de surveillance épidémiologique’s officers (CISSE), Direction régionale de la santé’s director (DRS), Infirmier chef de poste (ICP), District medical officers (MCD), Disease control service manager (SLM)

**Performance of the routine HIS: timeliness and completeness of data**

**Community reports transmitted from Districts to the central level:** data concerning the timeliness of the transfer of CHBW reports from the HDs to the central level were unavailable for the study period of 2016-2019 (Table 2). In terms of completeness of reports, there were no available data for the year 2016 while for the years 2017-2019, completion percentages were recorded without absolute numbers. In 2017, 7 of 15 HDs (47%) recorded 0% completeness of reports; 4 HDs achieved less than 9% completeness, while 4 HDs reported more than 80% completeness (Table 2). Three of the 4 'high' performance HDs maintained high completeness scores (>99%) in 2018, while 4 of the 7 'low' performance HDs remained in the lowest category (<0.1%). All other HDs recorded some or significant improvements in their completeness scores in 2018, with 1 HD changing from 0% completeness of CHBW reports in 2017 to 100% completeness in 2018. In 2019, all high-performance HDs recorded a noticeable drop, ranging between 45% and 62% completeness, while the remaining HDs recorded some improvement - though the completeness scores remained below 60% (Table 2).

**Table 2:** completion of community-based health workers reports data entry in Health Districts of Centre-East and Hauts Bassins regions from 2016 to 2019, Burkina Faso

		2016		2017		2018		2019	
Health region	Health district	Completeness		Completeness		Completeness		Completeness	
		N	%	N	%	N	%	N	%
**Centre-East**	Bittou	Not stated	-	Not stated	0.7	Not stated	33.5	Not stated	39.4
	Garango	Not stated	-	Not stated	0.0	Not stated	0.0	Not stated	60.0
	Koupèla	Not stated	-	Not stated	0.0	Not stated	0.1	Not stated	38.5
	Ouargaye	Not stated	-	Not stated	0.0	Not stated	0.0	Not stated	18.4
	Pouytenga	Not stated	-	Not stated	0.0	Not stated	0.1	Not stated	12.7
	Tenkodogo	Not stated	-	Not stated	2.4	Not stated	16	Not stated	42.3
	Zabré	Not stated	-	Not stated	82.8	Not stated	99.8	Not stated	45.6
**Hauts bassins**	Dafra	Not stated	-	Not stated	100	Not stated	99	Not stated	62.0
	Dandé	Not stated	-	Not stated	89.7	Not stated	64.7	Not stated	38.9
	Do	Not stated	-	Not stated	0.0	Not stated	43.5	Not stated	98.1
	Houndé	Not stated	-	Not stated	8.6	Not stated	50.6	Not stated	41.7
	Karangasso Vigué	Not stated	-	Not stated	93.8	Not stated	99.4	Not stated	57.3
	Léna	Not stated	-	Not stated	8.9	Not stated	11	Not stated	28.6
	Orodara	Not stated	-	Not stated	0.0	Not stated	19.8	Not stated	58.6
	N’Dorola	Not stated	-	Not stated	0.0	Not stated	100	Not stated	59.3

**Weekly official letter telegrams (TLOHs) transmitted from each health district to the central level:** apart from a single HD (Pouytenga) in 2017, all 15 HDs displayed high performance in terms of timeliness (>98%) and completeness (>98%) of TLOH transmission to the central level for the years 2016-2018 (Table 3). A noticeable lower performance was observed across all HDs in 2019, with both timeliness and completeness falling below 60%.

**Table 3:** performance of weekly official letter telegram (TLOH) transmission from 2016 to 2019 in Health Districts of Centre-East and Hauts Bassins regions, Burkina Faso

		2016			2017			2018			2019		
Health region	Health District	No. Reports	Timeliness^1^ (%)	Completeness^2^ (%)	No. Reports	Timeliness^1^ (%)	Completeness^2^ (%)	No. Reports	Timeliness^1^ (%)	Completeness^2^ (%)	No. Reports	Timeliness^1^ (%)	Completeness^2^ (%)
**Centre-East**	Bittou	624	100	100	572	100	100	572	100	100	620	56.6	56.6
	Garango	1464	100	100	1404	100	100	1404	100	100	1404	57.7	57.7
	Koupèla	1456	100	100	1485	100	100	1560	100	100	1560	57.7	57.7
	Ouargaye	1581	100	100	1508	100	100	1721	100	100	1813	56.2	56.2
	Pouytenga	1188	100	100	942	71	71	988	93.9	94.1	1132	57.2	57.2
	Tenkodogo	1715	100	100	1818	99.9	99.9	1820	99.3	100	1820	57.7	57.7
	Zabré	871	100	100	780	100	100	780	99.9	99.9	780	57.7	57.7
**Hauts bassins**	Dafra	884	99.7	100	884	100	100	884	100	100	884	57.7	57.7
	Dandé	1768	99.9	99.9	1767	100	100	1768	100	100	1769	57.7	57.7
	Do	1508	100	100	1508	100	100	1510	100	100	1508	57.7	57.7
	Houndé	1491	100	100	1667	100	100	1660	100	100	1770	58.6	58.6
	Karangasso Vigué	416	100	100	409	100	100	416	100	100	432	59.3	59.3
	Léna	778	100	100	780	98.3	98.3	820	100	100	832	57.7	57.7
	Orodara	1647	100	100	1849	100	100	1960	100	100	1961	57.4	57.4
	N’Dorola	797	100	100	882	99.9	99.8	884	100	100	884	57.7	57.7

1 Timeliness: percentage of expected reports delivered on time; ^2^Completeness: percentage of expected reports delivered during a given period

**Monthly activity reports (MARs) from health facilities were transmitted from each health district to the central level:** as in the case of community reports, timeliness data for MARs were unavailable across all years. All districts, except one (Do), recorded high completeness scores (>91%) for MARs in 2016 and 2018 (Table 4). In addition to Do, two HDs, recorded relatively low completeness scores (59.3% and 83.7%) in 2017, while all HDs recorded a drop in completeness scores in 2019, with scores ranging from 49% to 97.5%.

**Table 4:** completion of monthly activity reports of health facilities at the level of Health Districts in Centre-East and Hauts Bassins regions from 2016 to 2019, Burkina Faso

		2016		2017		2018		2019	
Health region	Health district and hospitals	Completeness		Completeness		Completeness		Completenes	
		N	%	N	%	N	%	N	%
**Centre-East**	*Centre hospitalier régional* (Tenkodogo)	120	100	168	100	168	100	168	68.5
	Bittou	132	100	132	100	121	100	144	55.6
	Garango	312	100	324	100	297	100	324	58.0
	Koupèla	336	100	360	100	371	100	384	57.0
	Ouargaye	348	99.7	360	100	364	100	420	57.4
	Pouytenga	216	100	216	59.3	204	100	228	75.9
	Tenkodogo	396	100	408	100	371	100	408	58.3
	Zabré	180	100	180	99.4	165	100	180	57.8
**Hauts bassins**	*Centre hospitalier universitaire* (Bobo)	252	100	252	100	276	100	276	73.9
	Dafra	300	91.3	300	83.7	111	100	312	49.0
	Dandé	408	100	408	100	376	100	408	59.1
	Do	612	75.8	600	73.8	542	84.5	612	66.2
	Houndé	372	100	396	100	361	100	432	97.5
	Karangasso Vigué	96	100	96	100	88	100	96	58.3
	Léna	180	100	180	100	176	100	192	58.3
	Orodara	408	100	444	100	418	100	456	80.3
	N’Dorola	180	100	204	100	188	100	204	61.8

**Strengths and weaknesses of the HIS: stakeholder perceptions**

**Technical factors: use of tools**

***Even if tools are available on the ground, some concerns remain related to the case definition, production and transmission of data***

Whereas standard case definitions were available at the PHCs, simplified case definitions for certain diseases were sometimes missing at the community level making it difficult for CBHWs to identify and report suspected cases: “*It would be a good thing for us if they could find more effective guides where all the definitions of diseases are included. [...] This will improve the work*” (I.Z, CBHW in Hauts bassins). At the hospital level, health workers also identified weaknesses in the notification of suspicious cases based on definitions: “*there are people who use case definitions [and] others who do not know case definitions, [...] so many cases escape us. Some use it, others [do not] use it because, well, they say [we are] in a hospital setting and it's not relevant for them [...] meaning that until we confirm, we can't declare the case. So here we are, they want to confirm first before declaring. Whereas in epidemiology you have to declare before you can confirm or refute after*” (D.D, member of a regional management team).

The production and transmission of CBHWs reports and monthly activity reports (MARs) experienced various challenges affecting the observed timeliness and completeness of reports from the HDs to the central level, even within the same region: “*When I take our region, the transmission of reports from CBHWs to PHCs is not done in the same way. There are some, which are regular, and some, which are not. Synthesis and transmission are the same things. Data entry on EnDoS is also the same*” (C.P. member of a regional management team). One explanation offered for the observed reporting irregularities across HDs included the uneven costs of data transfer: “*Some PHCs are not “floated ”. Therefore, we use our own credits to make calls. [...] If you take the 43 facilities here if we take out the ones that are “floated”, it is no more than 23 or 24, and the rest, if we need them, we must use our own units to make calls. However, there is no unit allocated for that*” (T.I, member of regional management team).

**Organizational factors: coordination and organization of surveillance activities**

***Behind the formal coordination of data management, some coping strategies take place:*** decision-making took place through formal instructions by email, or in a concerted/team approach with the District Management Team (DMT), provincial, regional committee, and regional health directorate (*Direction régionale de la santé* (*DRS*)) through social networks, with additional guidance (if needed) coming from the central level. But new tools of communication were also adopted “*[...] there is a growing use of applications such as WhatsApp, for exchanges between the DRS and district's health information and epidemiological surveillance officer* (Centre d´information sanitaire et de surveillance épidémiologique´s officer (CISSEs)).” (L.H, member of regional management team). However, some were skeptical about the efficient use of these new tools. “*[...] people tend to bring everything back to electronics and leave even paper behind. This is an important element, and [...] there must be, an archive of cases that should not be modifiable anymore. [...] Now that we are switching to electronic, how do we certify declarations? That remains another challenge*” (A.R, member of a regional management team).

***Local support and inter-sectoral collaboration needed:*** challenges were identified at the community level, due to the “compartmentalization” of sectors like health, education, and agriculture. A school teacher said: “*We always had this problem with the health workers [...] we don't work enough in tandem [...] I believe that if you hand the microphone to certain actors of education here, they risk saying the same thing to you. We even have the impression that there is a little conflict between us, and as long as the information goes from us to them, they do not come to us. That is it! Often [...] it is when you go for a consultation that you will find an old stock [of information poster] lying in the dust somewhere* (P.E. school teacher). The central level hesitated to delegate or to rely on local actors: “*How is it possible that the central level moves to the health facility level to perform activities? [...] The system is well designed; the system is known. If there is an activity in the field, the central level [could] call the District management team for training [...]. Then, the District calls the ICPs and trains them*” (B.W. member of a regional management team).

***The workload impact other activities:*** for health workers, the workload associated with weekly TLOH reporting had a negative impact on daily activities such as consultation, care, preventive, and promotional activities, whether at the PHC, *Centre medical* (CM/CMA) or the *Centre hospitalier regional/Centre hospitalier universitaire*(CHR/CHU) level. Health workers argued that CBHW reports and MARs were difficult to produce due to their detailed reporting requirements.

**Behavioural factors: information and decision-making dynamics:** the importance of data collection was recognized by all stakeholders across communities and facilities, but a number of constraints, such as a shortage of human resources and labor strikes, were mentioned by stakeholders. “*Logic would dictate that as soon as you are alerted by a CBHW that there is an unusual event happening in the village, you should immediately run to see because you don't know what it is. However, as you know a little bit, nowadays with resources becoming rare, health workers too...well...are not much in number. At times, the guy can be informed, but practically he can't [respond]. Either he is alone, he cannot leave patients who are already in the health facility and then go to investigate a rumor. So that means that some things pass in silence*” (K.M., member of regional management team in Hauts bassins). “*[...] Strikes in the last trimester of 2019 induced disruptions in the reporting of statistical data [...]. [This] situation has led to an adjustment of missing data using a robust statistical model [...]*” (Ministry of health).

Furthermore, treatment decisions for suspected cases were delayed due to an extensive time lag from sample collection to laboratory transfer, analysis and final feedback of results to relevant surveillance actors: “*It's long...it's long...it's long. It delays...and often even to send samples, people are not prompt...simply, because normally it [payment of the shipment] has to be refunded [...] but unfortunately, it is not done like that [...]. Therefore, [health workers] take samples and store them...They can wait a month before sending them*” (B.W., member of a regional management team in Haut bassins).

## Discussion

The performance of health information systems (HIS) occurs within an organizational setting with actors who need motivation, knowledge and skills to perform their tasks and where specialized technical know-how is required for timely analysis and reporting [17]. In countries with limited resources, the HIS often fails to offer guidance for performance improvements at the operational level [2]. Especially as the focus is often on data production rather than on action [18].

**Processes are affected by technical, organizational and behavioral factors:** our study shows that data collection and the flow of data through the HIS in Burkina Faso, seem to be out of step with the overall objectives of the system. Specifically, we observed low rates of timeliness and completeness for community and monthly activity reports from most of the health districts to the central level, with negative implications on the general performance of the system - not least on fundamental surveillance activities such as case identification and notification of diseases with epidemic potential. The inadequate reporting aligned with several weaknesses of the existing HIS as perceived by those directly involved in surveillance activities [17].

The flow of information from the CISSE to the HIS encountered several difficulties such as lack of or late data entry. It remains uncertain whether the low completion of CBHWs reports (Table 2) reflects the true absence of cases or if the data generated in the communities are irrelevant to surveillance activities. Indeed, the 2020 MEASURE evaluation in the Centre-South region of Burkina Faso reported that only 67 percent of center managers would be competent to use community-level or health facilities-level data [8]. This points to the importance of strengthening data quality and training. In the context of emerging epidemic diseases and given the important role of community actors in early disease detection, alert and response [19], actions and efforts should be discussed directly with the actors, taking the point of departure in the importance of data.

Furthermore, our data suggest that more emphasis should be placed on empowering the operational level which includes communities, PHC and CM/CMA, in line with WHO recommendations, by taking into account citizens' “expectations about health and health care” and ensuring “ that their voice and choice decisively influence the way in which health services are designed and operate” [20]. It is however essential that data flow from Districts is timely and complete.

In facilities, failure to use standard procedures during consultations by some health workers combined with a lack of measurement and monitoring of the performance of CHRs or CHUs may explain the current low performance in early detection of epidemics. Improved training and sensitization could alleviate this problem. The great potential of using CBHWs for social mobilization and for the implementation of community intervention could be strengthened. Notably, regular feedback from the central level to the community level must be integrated into the system, to stimulate and encourage the confidence of CHBWs in performing their tasks.

**Technical and organizational factors affect HIS process and performance directly or indirectly through behavioral determinants:** even if the HIS operates with standardized methods and a set of organizational rules and tools, it is essential that a sufficient number of staff is available to meet the workload. For health workers, HIS reporting represents a significant workload, and other time-consuming HIS activities include supervision of clinical activities by the ICP and investigation of disease rumors. This is in accordance with the study by Ndongo *et al*. in Cameroon, which found that health professionals responsible for data collection were discouraged by the large number of forms to be filled in due to the multiplicity of vertical programs [6]. Thus, these types of technical demands may lead to unproductive behavior such as long delays in reporting and low timeliness.

A major challenge in terms of confirming suspicious case lies in providing case definitions for all epidemic diseases in communities. Also, rapid detection and more complete laboratory data and case follow-up may avoid reporting delays, as lack of feedback and acknowledgment is perceived as highly demotivating. Full computerization of the HIS including integration of patient files could facilitate and accelerate the process of monitoring, storage, and extraction of case data. It would significantly reduce the time needed to process data at district, regional and national levels, while at the same time reducing the number of errors including the risk of data being compromised when transferred via informal routes, such as WhatsApp.

With managers at the intermediate and central levels, we observed an interest in improving the quality and integrity of data through ICT. The move toward the electronic system for integrated disease surveillance and response (e-IDSR), despite the skepticism of some HIS managers, seems to be the next step in disease surveillance [21]. However, in the context of limited financial resources and low priorities in the purchase and maintenance of computer equipment by districts, this could be a challenge. In addition, as stated by Aissi *et al*. [22], electronic management tools, based on technological innovations to improve performance have significant side effects such as stress and feelings of invasion of privacy among health staff [22]. In any case, it would be recommendable to focus HIS strengthening efforts on regular and systematic supervision visits at all levels of the health system, alongside a consensual development of reports and plans for the tracking and implementation of recommendations [8].

**Behavioral factors have a direct influence on performance:** in 2004 the insufficient use of existing data or information at the central level was criticized by the WHO [18]. More than 15 years later, efforts to decentralize health information and include patients and health workers in decision-making continue to be a challenge. Our results indicate that decision-making remains centralized, preventing the participation of peripheral structures with their local realities. In addition, our study shows that the motivation and capacity to search for suspected cases or for investigating rumors of potential outbreaks is low. Unnecessary delays are often created by health workers when transmitting samples to laboratories, with therapeutic and social implications for the patients, and such delays impede the early detection of epidemics.

The health workers' strike in Pouytenga in 2017, which resulted in the removal of 18 ICPs in the HD, as well as the major strike in 2019, had a notable effect on the HIS performance as shown in Table 3 and Table 4 [23,24]. Moreover, the 2019 strike for financial issues blocked the transmission of surveillance data on diseases with epidemic potential, especially during the 3^rd^ and 4^th^ quarters [12]. As mentioned by Phalkey *et al*., financial issues at any level directly affected logistics (vehicles, transport facilities, etc.), equipment availability as well as motivation. The latter in turn affect performance [25].

**Limitations:** we did not analyze the relevance of the implemented HIS, including its accuracy, the use of information and data, competencies in HIS tasks, and finances due to our methodology. These indicators would have required other more complex research tools and methods for their collection, analysis and assessment of the performance. Also, we did not have the possibility to assess the quality of the HIS reporting. Finally, we did not examine the quality of HIS, as we mainly focused on the stakeholder´s perceptions of how the system worked.

## Conclusion

Analysis of the performance of the HIS, based on the Routine Information System Management (PRISM) framework and from the point of view of stakeholders in Burkina Faso, makes it possible to identify and understand several weaknesses. We recommend that the strengthening of the health information management system over the next years be focused on the accuracy, updating, completeness, representativeness, relevance, complementarity of data and their appropriation by stakeholders and potential users. It is important to point out the possibility for the system to measure separate performances of the university and regional hospitals, and to share all CBHWs reports online, while applying the principle of One-health. Developing a culture characterized by regular use of available information for both operational action and strategic management and finally for a good performance is critical. New technologies, which offer opportunities for improvement should be tested in the local context, in order to explore opportunities to respond adequately to clearly defined public health objectives.

### What is known about this topic

- *HIS is a tool at the service of the health system, capable of improving its performance especially on data production;*
- *Burkina Faso's 2010-2020 national HIS strategic plan identified excessive and redundant data collection tools, weaknesses in data collection at the community level*.

### What this study adds

- *The HIS should include the possibility to measure individual performance of university and regional hospitals, and a complete share online of all CBHWs reports;*
- *The study shows that to improve the HIS performance, the capacity of CBHWs for social mobilization and for implementation of community intervention should be strengthened by associating actors in the community or the health system, with higher influence on social change and behaviour*.
